# Exploring Potential Diagnostic Biomarkers for Mechanical Asphyxia in the Heart Based on Proteomics Technology

**DOI:** 10.3390/ijms252312710

**Published:** 2024-11-26

**Authors:** Yuebing Huang, Hai Qiu, Qianling Chen, Zilin Meng, Dongfang Qiao, Xia Yue

**Affiliations:** Guangzhou Key Laboratory of Forensic Multi-Omics for Precision Identification, School of Forensic Medicine, Southern Medical University, Guangzhou 510515, China; yuebhuang@163.com (Y.H.); paqq251388201@163.com (H.Q.); singlingchan@163.com (Q.C.); 15815621217@163.com (Z.M.)

**Keywords:** mechanical asphyxia, proteomic, biomarkers, cause of death

## Abstract

Mechanical asphyxia presents a challenging diagnostic issue in forensic medicine due to its often covert nature, and the signs visible during an autopsy are usually not specific. Despite some progress in understanding hypoxia’s effects, traditional methods’ inherent limitations might overlook new biomarkers in mechanical asphyxia. This study employed 4D-DIA proteomics to explore the protein expression profiles of cardiac samples under conditions of mechanical asphyxia. Proteomic analysis identified 271 and 371 differentially expressed proteins in the strangulation and suffocation groups, respectively, compared to the control group. Seventy-eight differentially expressed proteins were identified across different mechanical asphyxia groups compared to the control group. GO and KEGG analysis showed enrichment in pathways, including complement and coagulation cascades, cAMP and cGMP-PKG signaling pathways, inflammatory mediator regulation of TRP channels, and phagosomes. Through stringent selection based on protein interactions, ALKBH5, NAA10, and CLPB were identified as potential diagnostic biomarkers. ALKBH5 showed increased expression in asphyxia models, while NAA10 and CLPB were downregulated; these biomarker changes were validated in both animal models and human cardiac samples. This study highlights the potential of proteomics in discovering reliable biomarkers, which can enhance the specificity of mechanical asphyxia diagnosis in forensic practice, provide new insights into the pathophysiological mechanisms of mechanical asphyxia, and offer new perspectives for diagnosing mechanical asphyxia.

## 1. Introduction

The diagnosis of mechanical asphyxia represents a challenging and significant matter in forensic medicine [1,2]. Currently, the diagnosis of mechanical asphyxia predominantly depends on meticulous autopsy procedures; however, the signs resulting from mechanical asphyxia are not specific, and numerous other causes of death may exhibit comparable manifestations [3,4,5]. Furthermore, such signs might even be absent in special types of mechanical asphyxia, such as infant asphyxia and postural asphyxia [6,7,8]. The diagnostic complexity of mechanical asphyxia also originates from its high degree of concealment. Many instances occur indoors or in concealed settings, and bodies are typically discovered belatedly [9,10]. Delays in discovery may obscure crucial signs, thereby obfuscating the cause of death. In such cases, the medical examiner can typically only speculate on “possible mechanical asphyxia”, and it is arduous to arrive at a definitive conclusion [11]. Nevertheless, cases of mechanical asphyxia are often closely associated with criminal activities and exert a profound influence on society and the judicial system [12,13]. Hence, the search for biomarkers that are specific and stable is particularly crucial in challenging cases where the corpse lacks distinct morphological evidence. Biomarkers not only furnish objective evidence for the identification of mechanical asphyxia but also offer significant diagnostic clues in complex cases where signs are ambiguous or absent. 

Previous researchers have made progress in the field of mechanical asphyxia based on prior knowledge through specific hypotheses or known biological issues. For instance, based on a general understanding of hypoxic effects, research has confirmed an increased expression of hypoxia-inducible factor 1-alpha (HIF-1α) in the pulmonary vessels of mechanical asphyxia [14]; furthermore, an in-depth understanding of endoplasmic reticulum stress has promoted the use of the stress-related protein CHOP in asphyxia diagnostics [15]. While these studies provide valuable insights, they are limited by the established knowledge system, which may overlook critical molecules and biological processes not yet extensively studied, potentially missing unknown markers closely related to mechanical asphyxia and introducing bias. Consequently, omics technologies, which are comprehensive and unbiased, are increasingly being utilized in medical and forensic fields to discover a range of biomarkers [16,17]. By employing untargeted metabolomics, researchers have identified metabolites like lactate, succinate, and xanthine in animal models as distinguishers between asphyxia and sudden cardiac death [18,19]. The metabolites typically have a molecular weight under 1500 Da, with many even smaller than 1000 Da [20]. Many small molecule metabolites are inherently unstable [21], influenced by enzymes and various chemical factors in the body, raising concerns about their stability post-collection [22,23,24]. Research shows that even short-term storage at room temperature before centrifugation (30 min) is enough to cause significant changes in metabolites, and the levels of xanthine increase under any storage condition, whether at room temperature or in the cold [25]. However, in practical forensic work, the discovery of bodies is often delayed due to the covert nature of mechanical asphyxia incidents. Thus, developing stable and reliable biomarkers for mechanical asphyxia is crucial, with proteins offering advantages over metabolites.

The 4D data-independent acquisition (DIA) proteomics technology, in contrast to traditional TMT and Label-Free methods, achieves nearly 100% precursor ion collection efficiency. This capability prevents the omission of low-abundance but biologically significant proteins, offering a more objective and comprehensive protein profile [26]. Proteomics, compared to transcriptomics and metabolomics, more directly mirrors the functional state of cells since proteins directly participate in executing most biological activities [27,28]. Moreover, proteomics in the analysis of biological processes is not limited by the non-one-to-one relationship between gene expression and protein function. Metabolomics also exhibits notable complexity, with a single metabolic precursor potentially converting into various metabolites via multiple pathways or several precursors synthesizing the same metabolite [29,30]. Moreover, the heart, with its negligible glycogen reserves and high oxygen demands, is extremely sensitive to ischemic and hypoxic conditions [31].

Consequently, to confront the challenges of mechanical asphyxia in forensic medicine and the limitations of traditional research approaches, in this investigation, we chose to adopt 4D-DIA proteomics technology within the heart to discern more stable biomarkers competent for diagnosing mechanical asphyxia. In this study, we showcased a more comprehensive protein landscape, identified the shared differentially expressed proteins (DEPs) in diverse types of mechanical asphyxia, and probed their potential biological functions via bioinformatics. Furthermore, to make the results as practical as feasible in the future, we formulated systematic screening criteria. Eventually, three potential biomarkers for the diagnosis of mechanical asphyxia were determined. These three potential biomarkers were validated at both the animal and human levels. These discoveries provide novel perspectives on the pathological mechanisms and biological processes related to mechanical asphyxia and introduce new strategies for its forensic diagnosis.

## 2. Results

### 2.1. Proteomic Analysis of Mechanical Asphyxia

**Figure 1:**
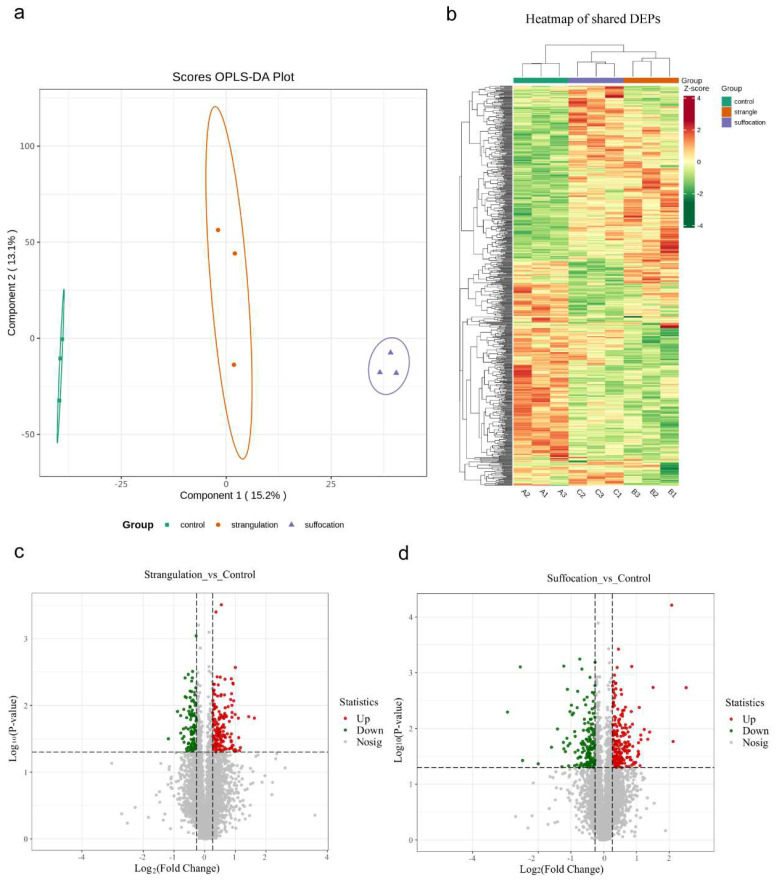
Proteomic analysis. (**a**) Significant separation between the control, strangulation, and suffocation groups in the OPLS−DA model. Each data point is corresponding to a myocardial protein sample derived from an individual animal. (**b**) Cluster analysis reveals distinct expression patterns among the three groups. (**c**,**d**) Volcano plots for the strangulation and suffocation groups compared to the control group. Upregulated DEPs are shown in red and downregulated in green.

4D-DIA proteomics analysis was conducted on the left ventricular myocardium of rats in the strangulation group, suffocation group, and control group, identifying and quantifying a total of 7557 proteins. Orthogonal partial least squares discriminant analysis (OPLS-DA) based on the proteomic data demonstrated effective separation between the control, strangulation, and suffocation groups (Figure 1a). Moreover, cluster analysis revealed distinct expression patterns differentiating the three groups (Figure 1b). Proteins with a fold change (FC) greater than 1.2 or less than 0.833 and a *p*-value lower than 0.05 were considered differentially expressed. Comparative analysis identified 271 differentially expressed proteins between the strangulation and control groups, with 160 proteins upregulated and 111 downregulated (Figure 1c). In the comparison between the suffocation and control groups, 371 proteins were differentially expressed, with 211 upregulated and 160 downregulated (Figure 1d).

### 2.2. Analysis of DEPs in Mechanical Asphyxia

**Figure 2:**
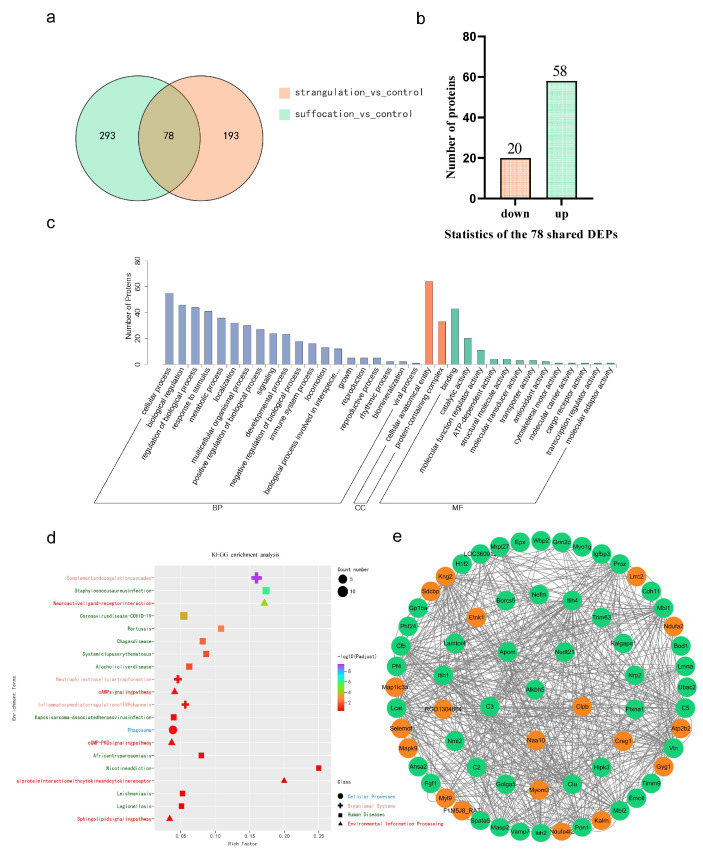
Bioinformatics analysis of shared DEPs. (**a**) A Venn diagram illustrates that there are 78 shared DEPs between the strangulation and suffocation groups compared to the control group. (**b**) Of the shared DEPs, 20 are downregulated, and 58 are upregulated. (**c**) GO annotation analysis categorizes the shared DEPs into molecular functions, biological processes, and cellular components. (**d**) KEGG enrichment analysis displaying the top 20 enriched pathways, where the *y*-axis shows pathway names and the *x*-axis shows rich factors. (**e**) PPI network analysis, with red indicating downregulated shared DEPs and green indicating upregulated shared DEPs.

In order to identify potential diagnostic biomarkers for various forms of mechanical asphyxia, a comparative analysis of DEPs across comparison groups was performed (Figure 2a), identifying 78 shared DEPs, with 58 being upregulated and 20 downregulated (Figure 2b). To investigate the functional features and biological information of the DEPs associated with mechanical asphyxia, GO annotation and KEGG enrichment analyses were conducted. GO annotation analysis indicated that the main biological processes (BPs) included cellular processes, biological regulation, metabolic processes, and responses to stimulus. The primary molecular functions (MFs) identified were binding, catalytic activity, and molecular function regulator activity. The cellular components (CCs) included cellular, anatomical entities, and protein-containing complexes (Figure 2c). KEGG enrichment analysis showed that the shared DEPs were enriched in pathways including the complement and coagulation cascades, cAMP signaling pathway, cGMP-PKG signaling pathway, inflammatory mediator regulation of TRP channels, and phagosomes (Figure 2d).

### 2.3. Screening for Potential Biomarkers for the Diagnosis of Mechanical Asphyxia

To further identify potential biomarkers for diagnosing mechanical asphyxia and to facilitate the comprehension of their interactions, a series of screening steps were established. First step: a protein interaction network analysis on 78 shared DEPs was conducted to investigate their interrelations (Figure 2e); Second step: those confirmed in the proteomics database with ‘experimental evidence at the protein level’ were selected; Third step: differentially expressed proteins showing homology in both animal and human samples were selected to improve the cross-species applicability of the markers; Fourth step: the Human Protein Atlas was utilized to select proteins with high expression levels in human cardiac tissues; Fifth step: prioritized proteins with greater differential expression fold changes were prioritized; Final step: Special attention was given to the functional proteins that are critical in regulating the structure and function of intracellular biomolecules. Through this comprehensive strategy, RNA demethylase ALKBH5, N-terminal acetyltransferase 10 (NAA10), and mitochondrial disaggregase (CLPB) were identified as candidate markers for mechanical asphyxia. ALKBH5 showed upregulation, whereas NAA10 and CLPB were downregulated in models of mechanical asphyxia.

### 2.4. Validation of Potential Biomarkers at the Animal Level

**Figure 3:**
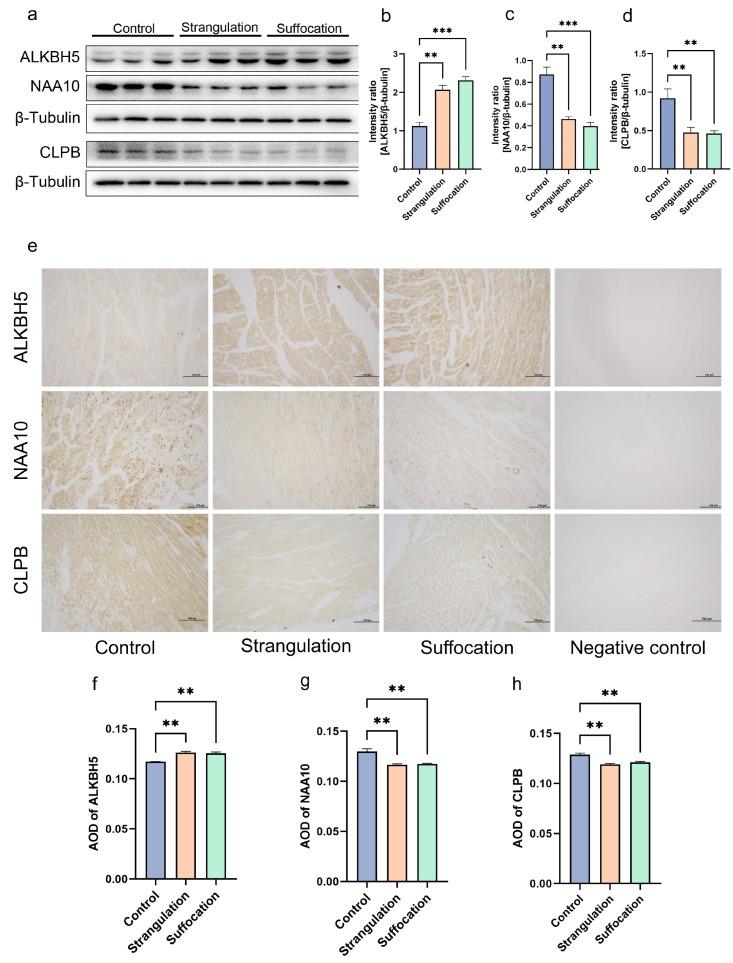
Validation of three potential biomarkers at the animal level by Western blot and immunohistochemistry. (**a**) Western blot analysis for ALKBH5, NAA10, and CLPB using β-Tubulin as the loading control, *n* = 3. (**b**–**d**) Relative expression levels in the strangulation, suffocation, and control groups. (**e**) Immunohistochemistry results, 200× magnification, The scale bar in the Figure is 100 μm (**f**–**h**) Statistical analysis of AOD values for the strangulation, suffocation, and control groups. ** denotes *p* < 0.01 and *** denotes *p* < 0.001.

Based on the 4D-DIA proteomics results, candidate biomarkers were validated in animal heart tissues through Western blot (WB) and IHC. WB analysis indicated a significant upregulation of ALKBH5 in the strangulation and suffocation groups and a significant downregulation of NAA10 and CLPB in the mechanical asphyxia group compared to the control (Figure 3a,b). These findings were consistent with the proteomics results. Furthermore, immunohistochemistry analysis showed that ALKBH5 was also significantly upregulated in the cardiac tissues of mechanical asphyxia cases, with trends for NAA10 and CLPB consistent with the Western blot results (Figure 3c,d).

### 2.5. Validation of Potential Biomarkers in Autopsy Samples

**Figure 4:**
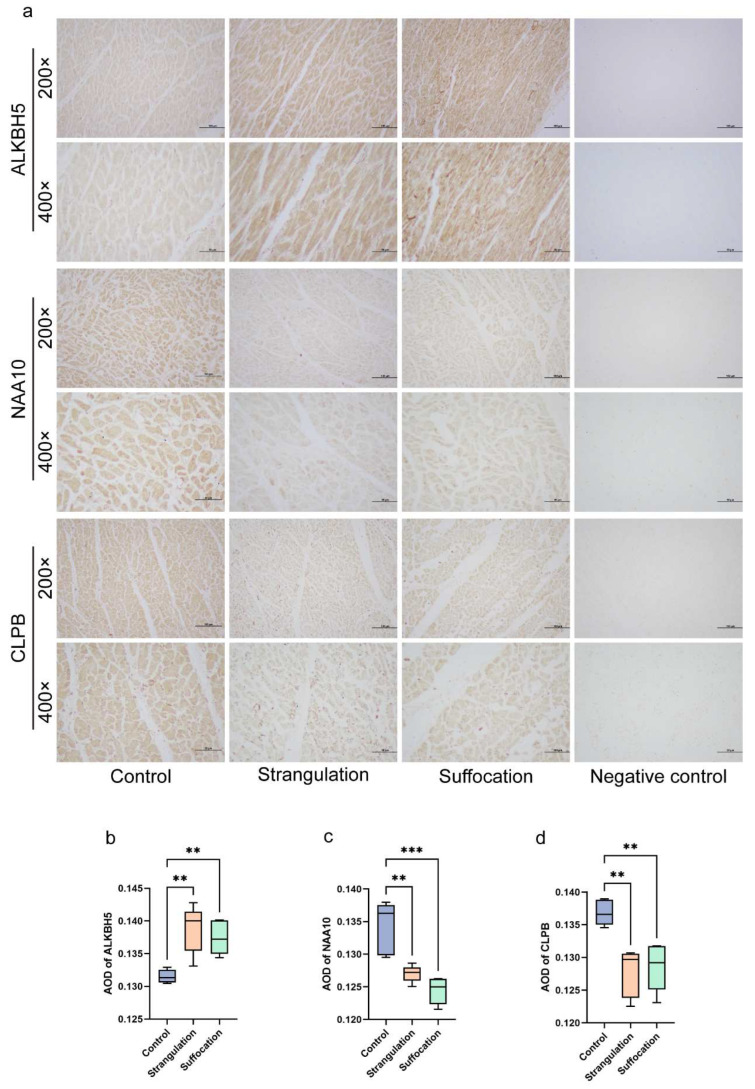
Immunohistochemical validation of three potential biomarkers in human samples. (**a**) Immunohistochemistry results; scale bars are 100 μm at 200× magnification and 50 μm at 400× magnification. (**b**–**d**) Quantitative analysis of the immunohistochemistry results, with ** indicating *p* < 0.01 and *** indicating *p* < 0.0001.

Immunohistochemical validation was performed for the expression of ALKBH5, NAA10, and CLPB in human heart tissues (Figure 4a). Compared to the group Fall from height, significant decreases in NAA10 and CLPB were observed in both mechanical asphyxia groups, whereas ALKBH5 demonstrated a significant increase in these groups. These findings align with the validation results observed in animal models.

## 3. Discussion

In forensic practice, the diagnosis of mechanical asphyxia is challenging due to the absence of specific signs, and even in some complex cases, such signs may not manifest [32,33]. Therefore, the development of targeted biomarkers is crucial for improving the accuracy of diagnosing mechanical asphyxia. In this study, we delineated the proteomic landscape of mechanical asphyxia using 4D-DIA proteomics, identifying 78 shared DEPs. Among these DEPs, based on the PPI network and specific criteria, we identified three potential biomarkers for diagnosing mechanical asphyxia. These markers were further validated in animal myocardial samples through the application of Western blot and immunohistochemistry techniques. Ultimately, these markers were validated in human samples as well, confirming their potential for immunohistochemical diagnosis of mechanical asphyxia in forensic practice.

In this study, we utilized 4D-DIA proteomics to analyze the left ventricular myocardium of various mechanical asphyxia groups compared to a control group, successfully identifying and quantifying 7557 proteins. Through comparative analysis, we discovered 78 shared DEPs. Based on GO annotation analysis, the BP of these shared DEPs encompassed cellular processes, biological regulation, metabolic processes, and responses to stimulus, with their MF predominantly involving binding, catalytic activity, and molecular function regulator activity to the heart’s stress response to mechanical asphyxia. In the KEGG enrichment analysis, the shared DEPs were predominantly enriched in pathways including complement and coagulation cascades, cAMP signaling, cGMP-PKG signaling, inflammatory mediator regulation of TRP channels, and phagosomes. The activation and regulation of these pathways likely interact through various mechanisms, collectively influencing the heart’s physiological response to mechanical asphyxia. Specifically, the activation of complement and coagulation cascades serves a dual role in mechanical asphyxia by initiating inflammatory responses and potentially exacerbating cardiac ischemia by promoting microvascular thrombosis [34,35], thus worsening cardiac injury. However, the cAMP and cGMP-PKG signaling pathways act protectively in cellular stress and energy metabolism regulation. These pathways help regulate glucose metabolism and fatty acid oxidation in cardiomyocytes, providing necessary energy support during mechanical asphyxia [36,37,38]. These processes are closely associated with the activation of the complement system and intracellular protein handling [39,40,41], demonstrating the complex interplay and synergistic effects of these pathways. Activation of the phagosome pathway aids in clearing damaged and apoptotic cells during the heart’s response to mechanical asphyxia and also influences the regeneration and repair of cardiac cells [42,43]. The regulation of TRP channels plays a crucial role in maintaining ionic balance and signal transmission in cardiac cells under hypoxic conditions [44,45], directly impacting cell survival. In summary, analyzing these pathways not only deepens our understanding of the impact of mechanical asphyxia on the heart but also may reveal approaches to optimize cardiac function and cell survival post-asphyxia through pathway modulation.

Through protein interaction analysis and strict selection criteria, ALKBH5, NAA10, and CLPB were identified as potential biomarkers for diagnosing mechanical asphyxia. Interactions among proteins are key to intracellular signaling and performing cellular functions [46,47]. Examining the interactions between shared DEPs unveils their roles in biological processes, specifically how they respond to mechanical asphyxia. This assists in identifying key proteins pivotal in mechanical asphyxia, directing subsequent validation of potential biomarkers and mechanistic research. Selecting proteins verified with ‘experimental evidence at the protein level’ in proteomics databases guarantees the detectability of selected biomarkers under contemporary experimental standards. Homologous proteins, by preserving structural and functional similarities across different species [48,49], enhance the generalizability of discoveries and the potential for translational research, ensuring that findings from mechanical asphyxia biomarker studies in animal models are applicable in forensic practices. Prioritizing proteins highly expressed in human cardiac tissues and with notable differential expression ensures their detectability in forensic contexts, improving diagnostic efficiency. Special attention is given to the functional proteins that are critical in regulating the structure and function of intracellular biomolecules. Under acute stresses such as mechanical asphyxia, synthesizing new proteins de novo in response is generally unfeasible due to the time-consuming nature of the process. Instead, biological processes that can be quickly modified, such as post-translational modifications, may be called for in this situation. Proteins participating in these processes maintain a high dynamic equilibrium under normal conditions and can undergo immediate changes in response to acute, life-threatening stresses such as mechanical asphyxia [50,51,52]. Focusing on these rapid dynamic regulatory mechanisms ensures the scientific soundness and responsiveness of the selected biomarkers in forensic applications. Our established screening criteria create a systematic process to pinpoint high-potential biomarkers from extensive datasets, offering a scientific and practical foundation for the diagnosis of mechanical asphyxia. This research design not only bolsters the scientific validity of the biomarkers but also enhances their practical use. ALKBH5 is one of only two known N6-methyladenosine (m6A) demethylases [53]. M6A is the most prevalent internal modification in eukaryotic mRNAs, playing crucial roles in various biological processes [54,55]. Under hypoxic conditions, m6A-modified RNA levels increase in cardiomyocytes and mouse hearts [56,57], highlighting m6A’s role in responding to physiological and pathological hypoxic stress. In our mechanical asphyxia model, ALKBH5 expression is significantly upregulated compared to the control group, consistent with the documented responses of cardiomyocytes to hypoxia. As a direct target of HIF-1α [58,59], ALKBH5’s upregulation in hypoxic conditions aids in regulating the expression of m6A-modified mRNA, fostering cellular adaptation to hypoxia and impacting cellular metabolism and survival [60]. Furthermore, a positive feedback mechanism exists between ALKBH5 and HIF-1α, which enhances adaptation to hypoxic conditions by improving glycolytic efficiency and HIF-1α activity [61]. ALKBH5 promotes anti-apoptosis and enhances the survival ability of cells under hypoxic conditions by upregulating the level of vascular endothelial growth factor A (VEGF-A) [62]. In cardiomyocytes, ALKBH5 also augments autophagic flux and inhibits cell apoptosis under hypoxia/reoxygenation conditions by reversing the m6A modification of TFEB mRNA [57]. Early in myocardial infarction, the response of ALKBH5 to hypoxia promotes scar repair [63] and improves cardiac function after infarction [64]. Based on the literature and our experimental results, the upregulation of ALKBH5 in mechanical asphyxia can be regarded as a stress response to hypoxia. It not only modulates metabolism and enhances anti-apoptotic mechanisms to cope with current hypoxic stress but also adjusts m6A modifications to alleviate the adverse effects of hypoxia. These findings emphasize the significant potential of ALKBH5 as a biomarker for diagnosing mechanical asphyxia.

In our study, NAA10 showed decreased expression under mechanical asphyxia conditions. NAA10, an acetyltransferase, is chiefly responsible for catalyzing protein acetylation in cells, affecting protein stability, subcellular localization, and interactions [65,66,67]. It is capable of regulating nutrient utilization and macromolecule circulation via acetylated phosphoglycerate kinase 1 (PGK1) under hypoxic conditions, facilitating cells’ adaptation to the low oxygen environment [68]. NAA10 also augments its capability to repair misfolded proteins by acetylating heat shock protein 70 (Hsp70), maintaining cell stability and enhancing survival under stress [69,70]. Additionally, NAA10 promotes HIF-1α degradation through direct binding to HIF-1α and increases HIF-1α-targeted proteasome degradation by inhibiting the HIF-1α factor (FIH) [71,72], suggesting that the downregulation of NAA10 could potentially be an adaptive response to hypoxia. The reduced expression of NAA10 may not merely reflect the requirement for metabolic regulation and protein repair under mechanical asphyxia but also might be associated with alterations in consumption or activity (alterations in antigenic epitopes, resulting in reduced detection). In summary, the changes in NAA10 expression provide direct evidence of the cellular physiological state under mechanical asphyxia conditions, reflecting significant changes in cellular adaptability and metabolic state and highlighting its potential as a diagnostic biomarker for mechanical asphyxia.

ATP production is reliant on mitochondrial protein homeostasis [73,74]. CLPB, an ATPase, maintains mitochondrial protein homeostasis by facilitating the proper folding and assembly of proteins within mitochondria [75]. Lack of CLPB results in mitochondrial cristae remodeling and stress responses, thereby triggering apoptosis [76]. In stress conditions such as hypoxia, an augmentation of protein misfolding and aggregation occurs within mitochondria, thereby inducing mitochondrial dysfunction [77,78]. During acute hypoxia in mechanical asphyxia, the homeostasis of mitochondrial proteins is disrupted, and the demand for CLPB may rise significantly in response to protein misfolding and aggregation. This excessive mobilization could result in its rapid depletion, ultimately being manifested by its decreased expression in the mechanically asphyxiated heart. This decline in expression reflects CLPB’s critical role in combating acute stress in the short term and may also point to adverse consequences of its long-term absence [75,79], highlighting its potential as a biomarker for mechanical asphyxia. Additionally, studies have shown that in E. coli, CLPB homologs interact with the DnaJ-recruited Hsp70 chaperone system to aid in protein disaggregation [80]. Whether CLPB also interacts with Hsp70 in humans remains to be further investigated, which could provide new insights into its role in mechanical asphyxia and responding to acute hypoxic stress.

In summary, this study utilized 4D-DIA proteomics to map the proteomic landscape of mechanical asphyxia, revealing that the shared differentially expressed proteins across mechanical asphyxia groups optimize cardiac function and cellular survival after asphyxia through pathways such as complement and coagulation cascades, cAMP signaling, cGMP-PKG signaling, inflammatory mediator-regulated TRP channels, and phagosome. Using the established systematic screening process, potential biomarkers for mechanical asphyxia were investigated. Validation at both animal and human levels confirmed that ALKBH5, NAA10, and CLPB are biomarkers with diagnostic potential for mechanical asphyxia. Our findings offer new insights into the pathophysiological mechanisms and biological processes associated with mechanical asphyxia, presenting fresh perspectives for its forensic diagnosis. However, our study has its limitations, as it assessed and utilized only three biomarkers, which may be insufficient for accurate diagnosis in complex cases. Additionally, the practical diagnostic efficacy of these results requires further validation with larger sample sizes and a broader range of death causes.

## 4. Materials and Methods

### 4.1. Animal Models and Specimen Collection

All experiments received approval from the Animal Ethics Committee of Southern Medical University (Approval No. SMUL202310023) and were carried out following the Guide for the Care and Use of Laboratory Animals. Male Sprague-Dawley (SD) rats (8 weeks old, SPF level) were obtained from the Guangdong Medical Laboratory Animal Center (Guangzhou, China). Following a week of acclimatization, 18 SD rats were randomly assigned into three groups (n = 6 per group). All experiments began post-anesthesia with sodium pentobarbital (30 mg/kg). In the strangulation group, rats were exterminated using a thin rope to apply external force [81]. The suffocation group utilized the model outlined by Hu Y et al. [15], where rats were asphyxiated in a transparent, sealed environment designed to mimic real-life mechanical asphyxia cases without obvious mechanical injuries. The control group comprised decapitated rats, whose rapid death process entails comparatively minor biochemical stress, thus more accurately reflecting the organism’s normal baseline. Sterile instruments were employed for sampling. The left ventricular myocardium, designated for proteomic and Western blot analyses, was swiftly frozen in liquid nitrogen and preserved at −80 °C until further use. The remaining samples were fixed in 4% paraformaldehyde and kept at 4 °C for subsequent histopathological examination.

### 4.2. Human Specimens

A total of fifteen cases were collected from the Forensic Science Center of Southern Medical University, with detailed information on the human samples provided in Table 1. Human samples were analyzed with the consent of the deceased’s relatives, adhering to the 1964 Helsinki Declaration. Cases were divided into three groups according to the cause of death, with each group comprising five samples. The first group consisted of individuals who died from high falls. Detailed investigations confirmed that all died immediately post-fall, without receiving medical intervention, serving as controls for the other groups. The second group’s cause of death was strangulation. The third group died from suffocation.

### 4.3. Proteomics

#### 4.3.1. Sample Processing

The left ventricular myocardium was ground into powder in liquid nitrogen. This was followed by the addition of lysis buffer containing 1 mM of PMSF and 2 mM of EDTA and incubated for 5 min. The sample was subjected to ultrasonication for 5 min. After centrifugation of the lysate (4 °C, 15,000× *g*, 20 min), the supernatant was collected. Total protein concentration was measured using a BCA protein assay. An equal volume of protein solution was taken based on protein concentration, and the volume was adjusted to 200 µL with lysis buffer, followed by DTT reduction (37 °C, 10 mM, 45 min) and iodoacetamide (IAM) alkylation (50 mM, in the dark, for 15 min). Pre-chilled acetone was added to precipitate the proteins (−20 °C, four times the volume of the protein solution, for 2 h). After centrifugation, the protein precipitate was air-dried and re-suspended in an ammonium bicarbonate solution (200 µL, 25 mM) and trypsin (3 µL) for overnight digestion at 37 °C. Following digestion, peptides from each sample were desalted using C18 columns, concentrated via vacuum centrifugation, and re-dissolved in 0.1% (*v*/*v*) formic acid.

#### 4.3.2. LC-MS/MS Assays

Liquid chromatography (LC) analysis was performed using a nano-flow nanoElute UHPLC system (Bruker Daltonics, Karlsruhe, Germany). About 200 ng of peptides were eluted through a reverse-phase C18 column (25 cm × 75 µm ID, 1.6 µm, Aurora Series with CSI, IonOpticks, Melbourne, Australia) at a flow rate of 0.3 µL/min over a period of 60 min. The chromatography column temperature was held steady at 50 °C. Mobile phases A and B consisted of 0.1% formic acid in water and 0.1% formic acid in acetonitrile, respectively. In the first 45 min, the gradient of mobile phase B increased from 2% to 22%, then to 35% over the next 5 min, reached 80% in the following 5 min, and was maintained at 80% for the last 5 min. The LC system was directly interfaced with a hybrid timsTOF Pro2 mass spectrometer (Bruker Daltonics, Karlsruhe, Germany) using a CaptiveSpray nano electrospray ionization source (CSI). The timsTOF Pro2 was operated in data-dependent parallel accumulation-serial fragmentation (PASEF) mode to establish appropriate acquisition windows for diaPASEF mode. The analytical gradient was set at 60 min, operating in positive ion mode. Parent ion scans ranged from 100 to 1700 *m*/*z*, with ion mobility from 0.7 to 1.4 V⋅s/cm^2^, ion dwell time of 100 ms, and nearly 100% ion utilization. The capillary voltage was set at 1500 V, with a dry gas flow of 3 L/min and a drying temperature of 180 °C. n DDA-PASEF acquisition mode, the parameters included 10 MS/MS scans with a total cycle time of 1.17 s, charge state range of 0–5, dynamic exclusion time of 0.4 min, target ion intensity of 10,000, ion intensity threshold of 2500, CID fragmentation energy of 42 eV, and isolation windows set to 2 for *m*/*z* below 700 and 3 for *m*/*z* above 700. The parameters for diaPASEF acquisition mode included a mass range of approximately 400–1200, mobility range of 0.7–1.4 V⋅s/cm^2^, mass width of 25 Da, mass overlap of 0.1, 32 mass steps per cycle, and 2 mobility windows, totaling 64 acquisition windows. The average cycle time was 1.8 s.

#### 4.3.3. Database Search and Quantification

DIA-NN (version 1.8.1) was utilized for database searching with the Libraryfree method, employing the uniprot-proteome_UP000002494_Rat_20220719.fasta database (46,069 sequences), and using deep learning parameters to predict a spectral library. Utilizing match-between-runs (MBR), a spectral library was created from the DIA data, which was then used to reanalyze the DIA data for protein quantification. Both precursor ions and proteins were filtered with a false discovery rate (FDR) of 1%.

#### 4.3.4. Bioinformatics Analysis

Comprehensive functional annotations for identified differentially expressed proteins encompass Gene Ontology (GO) classifications (http://geneontology.org/, accessed on 10 November 2023) and Kyoto Encyclopedia of Genes and Genomes (KEGG) pathways (https://www.genome.jp/kegg/, accessed on 10 November 2023.). The STRING database (https://string-db.org/, accessed on 29 November 2023) was utilized to perform protein–protein interaction (PPI) analysis, identifying interactions among all DEPs. Evidence for the existence of differentially expressed proteins was confirmed through the proteomics database (www.uniprot.org/, accessed on 29 November 2023), and DEP expression levels in human heart tissues were determined via the Human Protein Atlas (www.proteinatlas.org/, accessed on 29 November 2023).

### 4.4. Western Blotting

Protein concentrations were measured using the Bradford protein assay kit (Beyotime, Shanghai, China). Protein lysates (20 μg) were separated on SDS-polyacrylamide gels and transferred onto PVDF membranes (Millipore, Billerica, MA, USA). Membranes were blocked for 2 h at room temperature using TBST buffer containing 5% non-fat milk. Incubated overnight with primary antibodies at 4 °C. Following this, membranes were incubated with secondary antibodies for 2 h at room temperature. Visualization was conducted using an ECL detection system (Tanon5200, Shanghai, China). Post-visualization of CLPB, membranes were stripped using primary and secondary antibody stripping solution (Beyotime, Shanghai, China) and then re-probed with a loading control antibody. Bands were semi-quantitatively analyzed using ImageJ software (version 1.54g, National Institutes of Health, Bethesda, MD, USA). Primary antibodies used included: (a) beta-Tubulin (1:10,000; Affinity, Lingyang, China); (a) ALKBH5 (1:5000; Proteintech, Wuhan, China); (a) NAA10 (1:3000; Proteintech, Wuhan, China); (d) CLPB (1:1000; Proteintech, Wuhan, China).

### 4.5. Immunohistochemistry Techniques

Following dewaxing, sections were rehydrated through a gradient and underwent high-temperature antigen retrieval for 10 min in 0.01 M sodium citrate buffer (pH 6.0, AR0024, BOSTER, Pleasanton, CA, USA) using a microwave, a process repeated twice. Tissues on slides were sealed with an immunohistochemical pen, then blocked as per the SP Rabbit and Mouse HRP Kit (CW2069, CWBIO, Cambridge, MA, USA), washed, and incubated overnight at 4 °C with diluted primary antibodies: ALKBH5 (1:200, Proteintech, Wuhan, China), NAA10 (1:200, Proteintech, Wuhan, China), and CLPB (1:200, Proteintech, Wuhan, China). Following DAB staining using the kit’s guidelines, sections were gradient dehydrated, cleared with xylene, mounted in neutral resin, and inspected under a microscope. In each Immunohistochemistry (IHC) procedure, a dilution of the primary antibody served as a surrogate for the specific antibody in the negative control.

For IHC quantification, a microscopy imaging system (Leica DM500, Wetzlar, Germany) was employed to randomly select five microscopic fields at or below 200× magnification from each section for analysis using ImageJ software (version 1.54g, National Institutes of Health, Bethesda, MD, USA). The staining intensity for each area is given as the total Integrated Optical Density (IOD), which is calculated by summing and excluding non-tissue areas within the field of view, reflecting the degree of positivity in the tissue regions. To reduce the influence of tissue area on the total IOD value, we utilize the Average Optical Density (AOD) as the final metric, calculated as the total IOD divided by the tissue area.

### 4.6. Statistical Analysis

Data are presented as mean ± SEM. A one-way ANOVA was conducted on all experimental data using GraphPad Prism software version 9.0 (GraphPad Software, Inc., La Jolla, CA, USA). Data were considered significantly different at *p* < 0.05.

## Figures and Tables

**Table 1:** Information of human samples.

Case No.	Gender	Age	PMI	Toxicology Screening	Cause of Death
Group 1					
1	Male	22 y	12 d	Negative	Fall from height
2	Male	/	3 d	Negative	Fall from height
3	Male	25 y	5 d	Negative	Fall from height
4	Female	32 y	4 d	Negative	Fall from height
5	Male	30 y	5 d	Negative	Fall from height
Group 2					
6	Male	49 y	3 d	Negative	Strangulation
7	Male	24 y	7 d	Negative	Strangulation
8	Male	40 y	8 d	Negative	Strangulation
9	Female	27 y	10 d	Negative	Strangulation
10	Male	37 y	10 d	Negative	Strangulation
Group 3					
11	Male	2 y	2 d	Negative	Suffocation
12	Male	28 y	10 d	Negative	Suffocation
13	Male	24 y	10 d	Negative	Suffocation
14	Female	2 m	1 d	Negative	Suffocation
15	Male	4 d	8 d	Negative	Suffocation

PMI, post-mortem interval.

## Data Availability

The raw data supporting the conclusions of this article will be made available by the authors on request.
